# Involvement of Rare Mutations of SCN9A, DPP4, ABCA13, and SYT14 in Schizophrenia and Bipolar Disorder

**DOI:** 10.3390/ijms222413189

**Published:** 2021-12-07

**Authors:** Chia-Hsiang Chen, Yu-Shu Huang, Ting-Hsuan Fang

**Affiliations:** 1 Department of Psychiatry, Chang Gung Memorial Hospital-Linkou, Taoyuan 333, Taiwan; yushuhuang1212@gmail.com; 2 Department and Institute of Biomedical Sciences, College of Medicine, Chang Gung University, Taoyuan 333, Taiwan; genie.cgu@gmail.com; 3 School of Medicine, College of Medicine, Chang Gung University, Taoyuan 333, Taiwan

**Keywords:** schizophrenia, bipolar disorder, whole-genome sequencing, rare mutation

## Abstract

Rare mutations associated with schizophrenia (SZ) and bipolar disorder (BD) usually have high clinical penetrance; however, they are highly heterogeneous and personalized. Identifying rare mutations is instrumental in making the molecular diagnosis, understanding the pathogenesis, and providing genetic counseling for the affected individuals and families. We conducted whole-genome sequencing analysis in two multiplex families with the dominant inheritance of SZ and BD. We detected a G327E mutation of SCN9A and an A654V mutation of DPP4 cosegregating with SZ and BD in one three-generation multiplex family. We also identified three mutations cosegregating with SZ and BD in another two-generation multiplex family, including L711S of SCN9A, M4554I of ABCA13, and P159L of SYT14. These five missense mutations were rare and deleterious. Mutations of SCN9A have initially been reported to cause congenital insensitivity to pain and neuropathic pain syndromes. Further studies showed that rare mutations of SCN9A were associated with seizure and autism spectrum disorders. Our findings suggest that SZ and BD might also be part of the clinical phenotype spectra of SCN9A mutations. Our study also indicates the oligogenic involvement in SZ and BD and supports the multiple-hit model of SZ and BD.

## 1. Introduction

Schizophrenia (SZ) and bipolar disorder (BD) are two devastating chronic mental disorders, with the pathogenesis remaining to be elucidated. Genetic factors play a significant role in the etiology of these two disorders [1,2]. The genetic bases of these two disorders are complex, involving common variants with modest effects and rare mutations with high clinical penetrance in multiple genes. Identifying common variants and rare mutations associated with SZ and BD has increased our understanding of the neurobiology of these two disorders [3,4,5]. Although SZ and BD are two psychiatric diagnoses from the clinical aspect, there is a significant overlap of symptoms between these two disorders [6]. It is not uncommon to observe members affected with SZ or BD within the same family. Furthermore, genetic studies showed overlaps of genetic variants between these two disorders, indicating these two disorders share some common heritability [7,8,9,10].

Rare genetic mutations associated with SZ and BD are highly heterogeneous, including chromosomal abnormalities [11], copy number variations (CNVs), and rare single-nucleotide variants [12,13]. Additionally, rare mutations associated with SZ and BD are highly personalized; most are private to the affected individuals and families. Identifying private pathogenic mutations associated with SZ and BD can help establish the molecular diagnosis, understand the pathogenesis, and provide helpful genetic counseling for the affected patients and families. Recent advances in high-throughput genome-wide mutation scanning methods, such as chromosomal microarray analysis (CMA) [14] and next-generation sequencing (NGS) [15], have facilitated the detection of private mutations associated with SZ and BD.

Rare pathogenic mutations associated with SZ and BD may occur sporadically or transmit within families. Family-based analysis can help elucidate the inheritance of rare mutations and their relationship with SZ and BD. Hence, identifying mutations cosegregating with psychiatric conditions in affected members within the family is an effective strategy to discover high penetrant pathogenic mutations associated with psychiatric disorders [16]. In our genetic study series of psychiatric disorders, we consecutively recruited families with single or multiple patients to search for their genetic deficits using systematic genetic analyses, including karyotype analysis, CMA, and NGS analysis.

## 2. Results

We recruited two multiplex families with SZ and BD transmitted in a dominant inheritance pattern. We did not find any chromosomal abnormality or pathogenic CNV in these two multiplex families. Nevertheless, whole-genome sequencing (WGS) analysis identified several rare deleterious missense mutations cosegregating with psychiatric conditions in these two families.

### 2.1. Family 1

**Figure 1:**
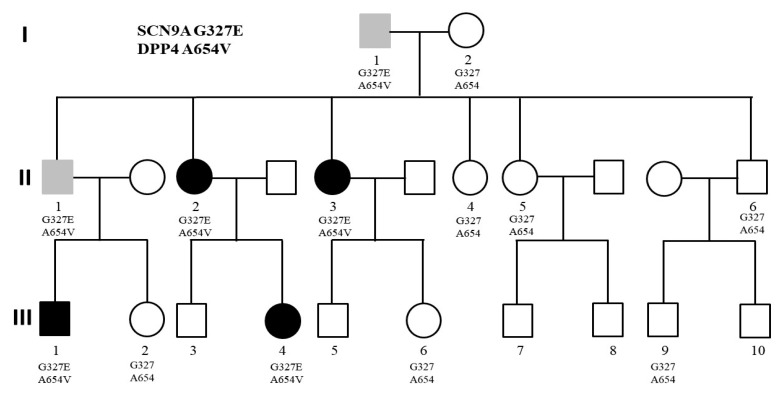
Pedigree of the three-generation family with multiple affected members. Black color indicates the diagnosis of SZ, while gray color indicates the diagnosis of BD. All the affected members carried the G327E mutation of SCN9A and the A654V mutation of DPP4.

The first family was a three-generation family with several members diagnosed with SZ and BD. As shown in Figure 1, the grandfather (I-1) was diagnosed with BD. He had six children, the first son (II-1) also had BD, but his two daughters (II-2 and II-3) were diagnosed with SZ. His other three children did not have mental disorders (II-4, II5, and II-6). Further, he had two grandchildren diagnosed with SZ (III-1 and III-4). The other grandchildren (III-2, III-3, III-5, III-6, III-7, III-8, III-9, and III-10) did not have any mental illness.

**Figure 2:**
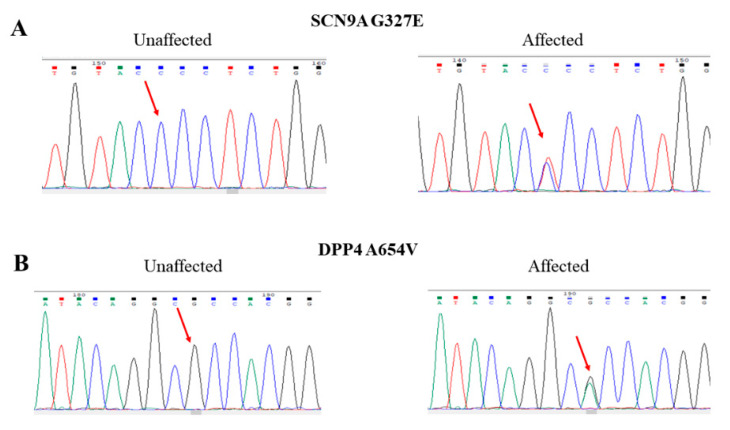
Representative Sanger sequencing tracings of the wild-type and heterozygous mutations of the G327E mutation of SCN9A (**A**) and the A654V mutation of DPP4 (**B**) in unaffected and affected family members, respectively.

We analyzed the whole-genome sequencing data from available family members under the dominant inheritance and identified two missense mutations cosegregating the psychiatric conditions in this family. We verified the authenticity of these two mutations using Sanger sequencing (Figure 2). The first one was a C-to-T change at nucleotide position 167149868 of chromosome 2 (rs765818027). The C-to-T change led to an amino acid sequence alteration from glycine to glutamic acid at codon 327 of the SCN9A gene, designated G327E. Bioinformatics analysis showed that the allele frequency of this mutation was very rare in several public genome databases, including Taiwan Biobank (Table 1). Additionally, several online software predicted that this mutation was pathogenic (Table 1).

The second mutation was a G-to-A change at nucleotide position 162865098 of chromosome 2 (rs149643982). The nucleotide change resulted in an amino acid substitution from alanine to valine at codon 654, designated Ala654Val. Bioinformatics analysis indicated that the allele frequency of this mutation was very rare in several public genome databases, including Taiwan Biobank (Table 1). Additionally, online software predicted this mutation had a deleterious effect on the DPP4 gene (Table 1).

### 2.2. Family 2

**Figure 3:**
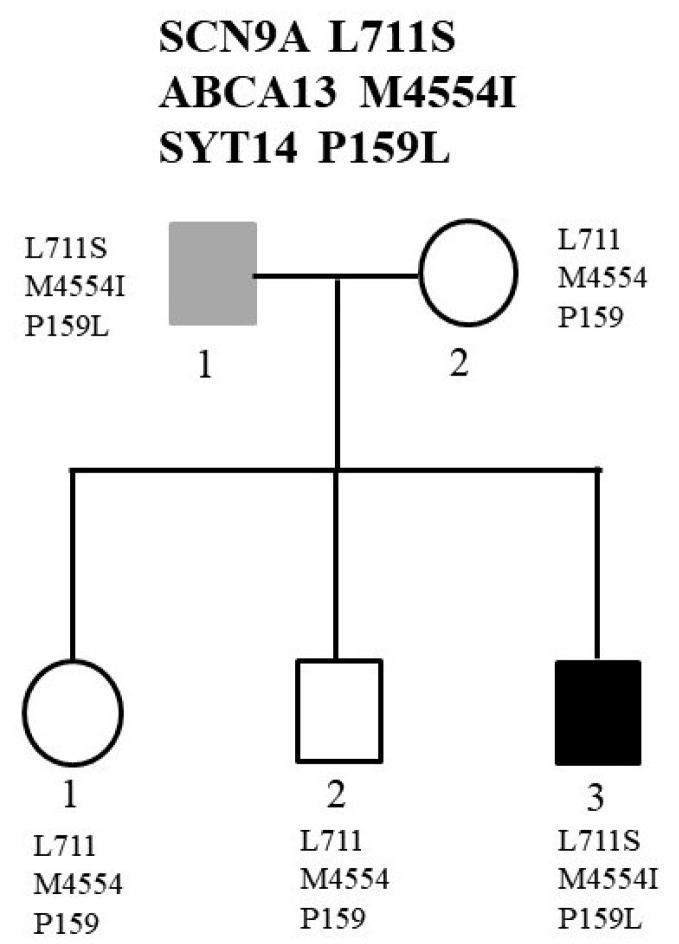
Pedigree of the two-generation family with the affected father and his son. Black color indicates the diagnosis of SZ, while gray color indicates the diagnosis of BD. The two affected members carried the L711S mutation of SCN9A, the M4554I mutation of the ABCA13, and the P159L mutation of the SYT14.

**Figure 4:**
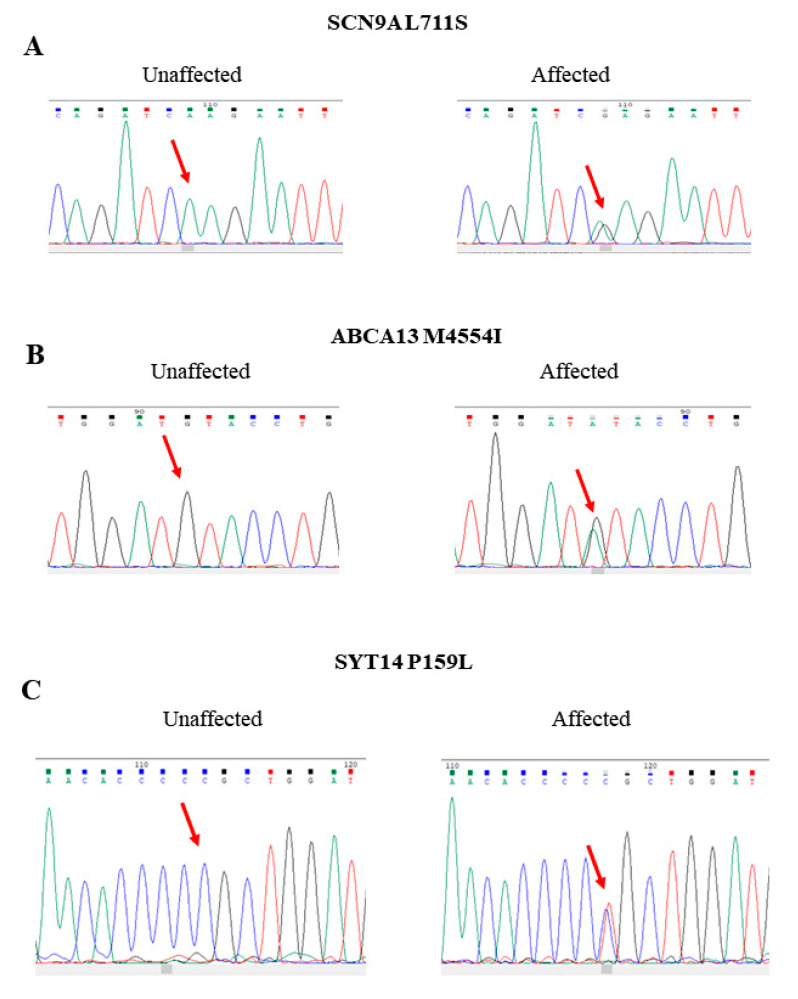
Representative Sanger sequencing tracings of the wild-type and heterozygous mutations of the L711S mutation of SCN9A (**A**), the M4554I mutation of the ABCA13 (**B**), and the P159L mutation of the SYT14 (**C**) in unaffected and affected family members, respectively.

The second family was a two-generation family consisting of five members. As shown in Figure 3, the father (II-1) was diagnosed with BD. He had three children. His third child was diagnosed with SZ (II-3), while the other two children (II-1 and II-1) did not have any mental disorders. We analyzed the WGS data under the dominant inheritance and detected three missense mutations cosegregating with the psychiatric diseases. The authenticity of these three mutations was verified by Sanger sequencing (Figure 4). The first mutation was an A-to-G change at the nucleotide position 167137045 of chromosome 2 (rs187526567). This nucleotide change led to amino acid substitution from leucine to serine at codon 711, designated Leu711Ser. The allele frequency of this mutation was very rare in several public genome databases, including Taiwan Biobank (Table 1). Online software also predicted that this mutation was pathogenic (Table 1).

The second mutation was a G-to-A substitution at nucleotide position 48556342 of chromosome 7. This substitution altered the amino acid sequence from methionine to isoleucine at codon 4554 of the ABCA13 gene, designated Met4554Ile. The allele frequency of this mutation was very rare in several public genome databases, including Taiwan Biobank (Table 1). Additionally, this mutation was predicted to have a damaging effect on the ABCA13 (Table 1).

The third mutation was a C-to-T substitution at the nucleotide position of 210267700 of chromosome 1 (rs77686387). This substitution altered the amino acid proline to leucine at the codon 159, designated Pro159Leu SYT14 gene. This mutation was also very rare in several public genome databases (Table 1). Additionally, online software predicted this mutation had a damaging effect on the SYT14 (Table 1).

## 3. Discussion

This study identified two rare mutations, the G327E mutation of SCN9A and the A654V mutation of the DPP4, cosegregating with SZ and BD in the first family and three rare mutations cosegregating with SZ and bipolar in the second family, including L711S mutation of SCN9A, M4554I mutation of ABCA13, and P159L of SYT14. Notably, each family had a missense mutation of SCN9A, respectively.

The SCNA9A gene encodes the sodium voltage-gated ion channel alpha subunit 9, which plays an essential role in nociception signaling. Loss-of-function mutations of SCN9A were associated with congenital insensitivity to pain [17], while gain-of-function mutations of this gene were associated with neuropathic pain syndromes, including erythromelalgia, paroxysmal extreme pain disorder, and small-fiber neuropathy [18,19]. In addition to pain-related diseases, mutations of SCN9A were associated with epilepsy in some case reports [20,21]. Nevertheless, a recent study reported no association between SCN9A and monogenic epilepsy disorders [22]. Of notice, the study by Robinson et al. reported that rare mutations of SCN9A were associated with autism spectrum disorders (ASDs). Their study found a C1143F mutation in the second intracellular loop of SCN9A shared in a family with multiple affected members. Furthermore, they found a significant increase of rare mutations in the second intracellular loop of SCN9A in ASD patients compared to control subjects. Hence, they concluded that rare mutations of SCN9A were involved in the etiology of autism in some families [23]. Together, these studies suggest that mutations of SCN9A might have pleiotropic clinical effects and play a role in neurodevelopmental disorders in addition to pain-related disorders.

The G327E mutation of SCN9A identified in the first family was located at the first extracellular loop of the sodium voltage-gated ion channel alpha subunit 9. Two previous studies found the G327E mutation in patients with various seizure phenotypes [21,24]. The L711S mutation of SCN9A in our second family was located at the first intracellular loop of the sodium voltage-gated ion channel alpha subunit 9, a novel mutation not reported associated with any disease in the literature to our knowledge. Our patients did not manifest symptoms related to pain or seizure. We propose that these two SCN9A mutations contribute to the pathogenesis of psychiatric conditions in these two families, and the SCN9A is a risk gene for SZ and BD. Our findings expand the clinical phenotype spectrum of SCN9A mutations.

In the first family, we detected another mutation, A654V of DPP4 cosegregating with psychiatric conditions. The DPP4 encodes the dipeptidyl peptidase 4, which is a multifunctional transmembrane protein expressed in various tissues. Altered concentration or activity of DPP4 in the blood was observed in patients with autism spectrum disorders [25,26], anxiety [27], depression [28], and eating disorders [29], suggesting DPP4 dysfunction is involved in various psychiatric disorders. To our knowledge, no mutation of DPP4 was reported to be associated with SZ and BD in the literature. Our study suggests that mutations of DPP4 contribute to the pathogenesis of SZ and BD in some patients.

We detected two additional mutations cosegregating with psychiatric conditions in the second family, the M4554I mutation of ABCA13 and the P159L of SYT14. The ABCA13 is a member of the ATP-binding cassette (ABC) gene superfamily consisting of seven subfamilies (from A to G) and at least 48 genes. The ABC family genes encode transporters that utilize the hydrolysis of ATP as the energy source to transport the substrates across the membrane to maintain the normal physiological functions in various tissues [30]. The ABCA13 encodes a large protein of 5058 amino acids and internalizes cholesterol and gangliosides from the plasma membrane to intracellular vesicles [31]. Knight and colleagues were the first to report the significant association of rare mutations of ABCA13 with SZ, BD, and major depression [32]. However, the other groups did not support this association [33,34]. The discrepancy might be due to the low frequency of ABCA13 mutations in patients with these psychiatric disorders or the different populations in their studies. The detection of the M4554I mutation of ABCA13 in this study agrees with the findings from Knight and colleagues and supports the proposal that rare mutations of ABCA13 might be involved in the pathogenesis of SZ, BD, and major depression [32].

The SYT14 gene encodes the synaptotagmin 14 protein, a member of the synaptotagmin gene family consisting of 17 members. Synaptotagmins regulate the membrane fusion of synaptic vesicles during neurotransmitter release [35]. They are calcium sensors and trigger the fast neurotransmitter release neurotransmission upon coupling the calcium influx [36]. The SYT14 is a calcium-independent synaptotagmin [37], expressed in the brain and other tissues [37,38]. Disruption of the SYT14 by a balanced chromosomal translocation t(1;3)(q32.1;q25.1) was reported in a girl with cerebral atrophy, macrocephaly, seizures, and developmental delay [38].

Additionally, Doi and colleagues reported a homozygous missense mutation of the SYT14 in a Japanese family with psychomotor retardation and cerebellar ataxia [39]. The mutation was a G-to-A substitution at the SYT14 cDNA nucleotide position 1451, which led to the amino acid change from the glycine to aspartic acid at codon 484, designated G484D. Together, these studies indicate that dysfunction of SYT14 might be involved in neurodevelopmental disorders. Hence, the P159L mutation of SYT14 identified in the second family might also contribute to the pathogenesis of SZ and BD.

Our findings in this study also support the oligogenic or multigenic involvement of SZ and BD [13]. Emerging evidence suggests that combinations of multiple rare mutations are an essential model of psychiatric disorders. For example, Kerner and colleagues reported detecting eight rare mutations shared by three affected siblings with BD in a family [40]. Goes and colleagues detected 84 rare damaging variants segregating with BD in eight multiplex families [41]. Maaser and colleagues conducted whole-exome sequencing (WES) in fifteen patients with BD in two large multiplex families from Cuba; they identified a total of seventeen rare potentially damaging mutations in seventeen genes shared by the affected patients in two respective families [42]. Ganesh and colleagues performed exome sequencing in thirty-two patients from eight multiplex families with severe mental illnesses. They identified forty-two rare deleterious mutations, with an average of around five mutations per family. None of them was shared across different families [43]. John and colleagues recently reported five rare damaging mutations from five different genes shared by four affected members in a four-generation SZ family [44]. Together, these studies indicate that interactions of multiple rare deleterious mutations are parts of the SZ and BD landscape.

## 4. Materials and Methods

### 4.1. Subjects

All subjects were residents of Taiwan. We consecutively recruited families with single or multiple cases affected with SZ and BD into this study. The Review Board of Chang Gung Memorial Hospital-Linkou approved this study with Approval Number 201801385A3. Each participant signed the informed consent after a full explanation of this study. We interviewed each participant and reviewed their medical records to collect their clinical information. The psychiatric diagnosis was based on the diagnostic criteria of the DSM-5 (Diagnostic and Statistical Manual of Mental Disorder-5th edition). The blood sample from each participant was collected for genetic experiments.

### 4.2. Karyotyping and Chromosomal Microarray Analysis

Karyotyping analysis was performed to search for chromosomal abnormalities using the conventional G-banding method according to the method established in the laboratory. The chromosomal microarray analysis was conducted following the manufacturer’s protocol to search for pathogenic CNVs using Affymetrix Genome-Wide Human SNP Array 6.0 (Affymetrix, Santa Clara, CA, USA). The CMA experiment was performed at The National Genotyping Center (Academia Sinica, Taipei, Taiwan).

### 4.3. Whole-Genome Sequencing (WGS) Analysis

Paired-end whole-genome sequencing was performed using the Illumina NovaSeq6000 platform (Illumina, San Diego, CA, USA) to search for small insertions and deletions (indels) and single-nucleotide variants (SNVs). The experiment followed the standard protocols provided by the manufacturer. After a quality check, the raw sequencing data were aligned to the human reference genome build hg19/GRch37. Afterward, we used the SAMtools and Genome Analysis Toolkit to refine the local alignment and generate a variant calling file for each subject. Variants were further annotated, filtered, and analyzed under the dominant inheritance. We used the SeqsLab software (Atgenomics, Taipei, Taiwan) to perform these analyses.

### 4.4. Sanger Sequencing

We designed primer pairs to obtain amplicons that covered the mutation by polymerase chain reaction (PCR)-based Sanger sequencing to verify the authenticity of mutations identified from whole-genome sequencing analysis. In brief, 30 cycles of PCR were performed in a 20 mL mixture containing 100 ng DNA, 1 μM of each primer, 1X buffer, 0.25 mM of dNTP, and 0.5 U of Power Taq polymerase (Genomics, New Taipei City, Taiwan). An aliquot of the amplicon was purified and subjected to Sanger sequencing using the BigDye Terminator kit v3.1 (Applied Biosystems, Foster, CA, USA). The primer sequences, optimal annealing temperature, and size of each amplicon are summarized in Table 2.

### 4.5. Bioinformatics Analysis

We checked the allelic frequency of the mutations identified in this study in the dbSNP (https://www.ncbi.nlm.nih.gov/snp/ (5 December 2021) and the Taiwan Biobank (https://taiwanview.twbiobank.org.tw/index (5 December 2021). Additionally, we conducted in silico analysis to assess the functional impact of mutations identified in this study using three online software, including Polyphen-2 (http://genetics.bwh.harvard.edu/pph2/index.shtml (5 December 2021), SIFT (https://sift.bii.a-star.edu.sg/ (5 December 2021), and PROVEAN (http://provean.jcvi.org/index.php (5 December 2021).

## 5. Conclusions

This study combined the WGS and the family analysis and identified multiple rare pathogenic mutations in four genes cosegregating with SZ and BD in two multiplex families. Our findings expand the genetic and allelic heterogeneity associated with SZ and BD and support the multihit model of complex psychiatric disorders. Further functional studies of these mutations are needed to address mechanistic pathogenesis.

## Figures and Tables

**Table 1:** Position, allele frequency, and functional prediction of rare mutations identified in this study.

Family	Gene and dbSNP	Position of Mutation	Allele Frequency		Functional Prediction		
			Taiwan Biobank	ALFA	PROVEAN	PolyPhen-2	SIFT
1	SCN9A rs765818027	NC_000002.11:g.167149868C>T NM_002977.3:c.980G>A NM_002977.3:p.Gly327Glu	0.001648	0	Deleterious	Probably damaging	Damaging
1	DPP4 rs149643982	NC_000002.11:g.162865098G>A NM_001935.3:c.1961C>T NM_001935.3:p.Ala654Val	0.000659	0.000156	Deleterious	Probably damaging	Damaging
2	SCN9A rs187526567	NC_000002.11:g.167137045A>G NM_002977.3:c.2132T>C NM_002977.3:p.Leu711Ser	0.003955	0.000068	Deleterious	Probably damaging	Damaging
2	ABCA13 rs142532424	NC_000007.13:g.48556342G>A NM_152701.4:c.13662G>A NM_152701.4:p.Met4554Ile	0.001978	0	Deleterious	Possibly damaging	Damaging
2	SYT14 rs77686387	NC_000001.10:g.210267700C>T NM_153262.3:c.476C>T NM_153262.3:p.Pro159Leu	0.003652	0.000318	Neutral	Probably damaging	Damaging

ALFA: Allele Frequency Aggregator; PROVEAN: Protein Variation Effect Analyzer; PolyPhen-2: Polymorphism Phenotyping v2; SIFT: Sorting Intolerant From Tolerant.

**Table 2:** Sequences of primers, optimal annealing temperature (Ta), and amplicon size for PCR-based sequencing of mutations identified in this study.

Gene and SNP	Forward Primer Sequences	Reverse Primer Sequences	Ta	Size (bp)
SCN9A rs765818027	5′-CACCAGGTACATATGCCATTC -3′	5′-TCCTTATTCAATATTGTCCCCC-3′	60 °C	313
DPP4 rs149643982	5′-ACCCAGCCTTGCAAAATAGCAG-3′	5′-GGAAACTGCGACTCGCTTACCA-3′	60 °C	357
SCN9A rs187526567	5′-ATTGGGTGGTGTTCCATAGC-3′	5′-GCCTGACTGATTTGTATCTGG-3′	60 °C	275
ABCA13 rs142532424	5′-TCAGGGATTCACCCCAAGGTC-3′	5′-GATGGCTAGCAACCGGGGCAT-3′	60 °C	241
SYT14 rs77686387	5′-GTTGCCATCAATTTTTTGATCCAG-3′	5′-CTTGGACTGTTGCTGCAGTGGG-3′	60 °C	264

## Data Availability

The raw data are available upon request of the corresponding author.
